# Supplementary material for: Fate of dissolved black carbon in the deep Pacific Ocean
Source: Nat Commun. 2022 Jan 13;13:307. doi: 10.1038/s41467-022-27954-0 (PMC8758769; doi:10.1038/s41467-022-27954-0)
Supplement: Supplementary file 1 — Supplementary Information [file 41467_2022_27954_MOESM1_ESM.pdf]

Supplementary Information for
Fate of dissolved black carbon in the deep Pacific Ocean

Youhei Yamashita[1,2,*], Motohiro Nakane[2], Yutaro Mori[2], Jun Nishioka[3,2], Hiroshi Ogawa[4]
[1]*Faculty of Environmental and Earth Science, Hokkaido University, Sapporo, Japan*
[2]*Graduate School of Environmental Science, Hokkaido University, Sapporo, Japan*
[3]*Pan-Okhotsk Research Center, Institute of Low Temperature Science, Hokkaido University, Sapporo, Japan*
[4]*Atmosphere and Ocean Research Institute, The University of Tokyo, Kashiwa, Japan*
*yamashiy@ees.hokudai.ac.jp

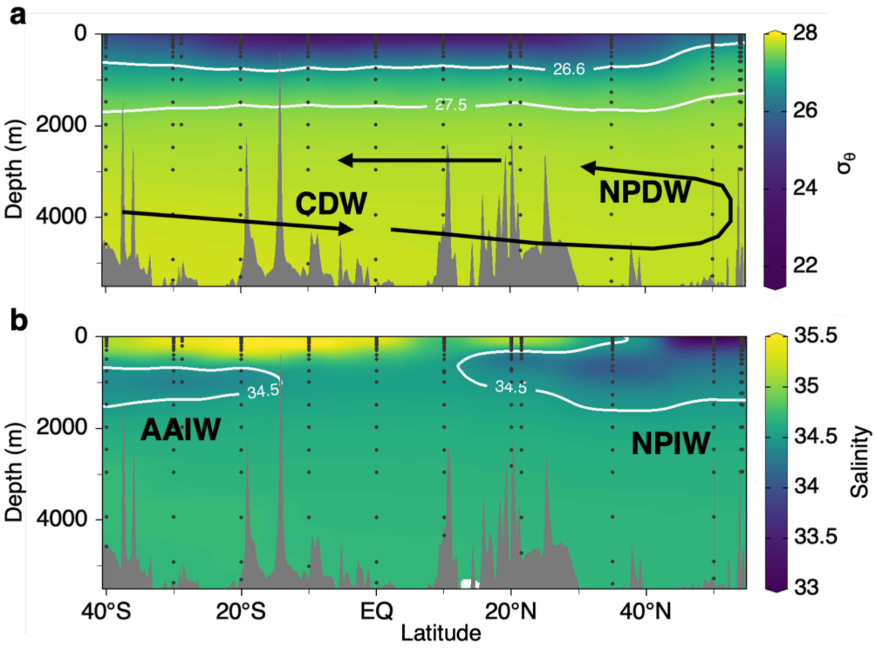

Supplementary Fig. 1. Meridional basin-scale distribution of potential density (σ_θ) and salinity. a, Distribution of potential density. The black arrows in the figure indicate the schematic path of deep-ocean meridional circulation in the Pacific Ocean, which is consistent with Circumpolar Deep Water (CDW) and North Pacific Deep Water (NPDW). **b**, Distribution of salinity. The white solid lines in the figure indicate salinity contours of 34.5 and correspond to Antarctic Intermediate Water (AAIW) and North Pacific Intermediate Water (NPIW). The sampling locations were described in Fig. 1a.
