# Supplementary material for: Fate of dissolved black carbon in the deep Pacific Ocean
Source: Nat Commun. 2022 Jan 13;13:307. doi: 10.1038/s41467-022-27954-0 (PMC8758769; doi:10.1038/s41467-022-27954-0)
Supplement: Supplementary file 3 — Description of Additional Supplementary Files [file 41467_2022_27954_MOESM3_ESM.pdf]

Description of Additional Supplementary Files

File name: Supplementary Data 1

Description: The dataset used in this study.
