# Supplementary material for: Fate of dissolved black carbon in the deep Pacific Ocean
Source: Nat Commun. 2022 Jan 13;13:307. doi: 10.1038/s41467-022-27954-0 (PMC8758769; doi:10.1038/s41467-022-27954-0)
Supplement: Supplementary file 5 — Reporting Summary [file 41467_2022_27954_MOESM5_ESM.pdf]

nature portfolio

Corresponding author(s): Youhei Yamashita

Last updated by author(s): Nov 24, 2021

Reporting Summary

Nature Portfolio wishes to improve the reproducibility of the work that we publish. This form provides structure for consistency and transparency in reporting. For further information on Nature Portfolio policies, see our Editorial Policies and the Editorial Policy Checklist.

Statistics

For all statistical analyses, confirm that the following items are present in the figure legend, table legend, main text, or Methods section.

n/a	Confirmed	
☐	☒	The exact sample size (n) for each experimental group/condition, given as a discrete number and unit of measurement
☐	☒	A statement on whether measurements were taken from distinct samples or whether the same sample was measured repeatedly
☐	☒	The statistical test(s) used AND whether they are one- or two-sided<br>*Only common tests should be described solely by name; describe more complex techniques in the Methods section.*
☒	☐	A description of all covariates tested
☒	☐	A description of any assumptions or corrections, such as tests of normality and adjustment for multiple comparisons
☐	☒	A full description of the statistical parameters including central tendency (e.g. means) or other basic estimates (e.g. regression coefficient) AND variation (e.g. standard deviation) or associated estimates of uncertainty (e.g. confidence intervals)
☐	☒	For null hypothesis testing, the test statistic (e.g. F, t, r) with confidence intervals, effect sizes, degrees of freedom and P value noted<br>*Give P values as exact values whenever suitable.*
☒	☐	For Bayesian analysis, information on the choice of priors and Markov chain Monte Carlo settings
☒	☐	For hierarchical and complex designs, identification of the appropriate level for tests and full reporting of outcomes
☒	☐	Estimates of effect sizes (e.g. Cohen's d, Pearson's r), indicating how they were calculated

Our web collection on statistics for biologists contains articles on many of the points above.

Software and code

Policy information about availability of computer code

Data collection	No software was used for data collection.
Data analysis	R (version 4.0.4) was used for scatter plots. Standard major axis regression and ordinary least squares regression were carried out using the lmodel2 package (version 1.7-3) in R (version 4.0.4). Ocean Data View (version 5.1.5) was used to produce the map and the basin-scale distribution of each parameter.

For manuscripts utilizing custom algorithms or software that are central to the research but not yet described in published literature, software must be made available to editors and reviewers. We strongly encourage code deposition in a community repository (e.g. GitHub). See the Nature Portfolio guidelines for submitting code & software for further information.

Data

Policy information about availability of data

All manuscripts must include a data availability statement. This statement should provide the following information, where applicable:

- Accession codes, unique identifiers, or web links for publicly available datasets
- A description of any restrictions on data availability
- For clinical datasets or third party data, please ensure that the statement adheres to our policy

The data that support the findings of this study are available in the Supplementary Data.

Field-specific reporting

Please select the one below that is the best fit for your research. If you are not sure, read the appropriate sections before making your selection.

☐ Life sciences ☐ Behavioural & social sciences ☒ Ecological, evolutionary & environmental sciences

For a reference copy of the document with all sections, see nature.com/documents/nr-reporting-summary-flat.pdf

Ecological, evolutionary & environmental sciences study design

All studies must disclose on these points even when the disclosure is negative.

Study description	We investigated meridional basin-scale distribution of dissolved black carbon (DBC) in the Pacific Ocean. We also measured dissolved oxygen (DO) concentration and calculated apparent oxygen utilization (AOU) as the difference between the saturated and measured DO concentrations. We found that DBC concentration is negatively correlated with AOU in the deep Pacific Ocean, implying that DBC is removed from the deep ocean to abyssal sediments through sorption onto sinking particles. We calculated the global flux of DBC removal through sorption onto sinking particles from the relationship between the DBC concentration and AOU with the global respiration rate in the open ocean.
Research sample	We collected seawater samples from the western and central Pacific Ocean by four cruises. The sampling sites and depths were selected to cover a wide range of water masses, and the samples can be considered representative of the water masses. The seawater samples were filtered and subjected to solid phase extraction (SPE). The SPE extracts were used to determine DBC concentrations and compositions with a benzenepolycarboxylic acid (BPCA) method. We also collected basic oceanographic data, i.e., temperature, salinity, and dissolved oxygen (DO) concentration, using a conductivity-temperature-depth (CTD) sensor with an oxygen sensor. These data were used to calculate AOU. The global respiration rate reported in a previous study was used to estimate the global flux of DBC removal.
Sampling strategy	To investigate the basin-scale distribution of DBC, seawater samples were collected from the surface layer to the deep layer with 12-L acid-cleaned, Teflon-coated Niskin-X bottles that were mounted on the CTD sensor with a carousel multisampling system during four cruises conducted in the Pacific Ocean, covering the area from 40°S to 54°N.
Data collection	Oceanographic data and seawater samples were collected by Y.Y., Y.M., J.N., and H.O. during R/V Hakuho Maru and R/V Professor Multanovskiy cruises. DBC was analyzed by M.N. and Y.M. under Y.Y.'s supervision using the BPCA method with high-performance liquid chromatography (HPLC) with a photodiode array detector (1260 Infinity, Agilent).
Timing and spatial scale	Four cruises were conducted in December 2013-January 2014, July 2014, September 2017, and August 2018. A large area of the Pacific Ocean (from 40°S to 54°N) was covered by the cruises.
Data exclusions	All measured data were included in this manuscript.
Reproducibility	Triplicate or duplicate samples were not collected over the observation because of limited amounts of seawater samples. However, the analytical error of the method was determined using triplicate seawater samples in our previous study and was <4% in terms of the DBC concentration.
Randomization	This is not relevant to our study because our field data was single.
Blinding	This is not relevant to our study, because we used in situ single data to determine the basin scale distribution of DBC.

Did the study involve field work? ☒ Yes ☐ No

Field work, collection and transport

Field conditions	Water temperature (potential temperature) ranged from 0.6 to 30.2°C. Salinity ranged from 32.4 to 36.2. AOU ranged from -39 to 311 μmol/kg.
Location	Thirteen stations conducted by four cruises covered longitude 162.99 to 190.30 and latitude -40.00 to 54.28. Depths from 0 to 5388m were covered.
Access & import/export	Oceanographic data were collected during four cruises. No permission is required by law to collect these data. The SPE cartridges for DBC analysis were stored frozen during the cruises and brought back to the laboratory on shore.
Disturbance	There is no disturbance because we carried out ship-based water sampling only.

Reporting for specific materials, systems and methods

We require information from authors about some types of materials, experimental systems and methods used in many studies. Here, indicate whether each material, system or method listed is relevant to your study. If you are not sure if a list item applies to your research, read the appropriate section before selecting a response.

Materials & experimental systems

n/a	Involved in the study
☒	☐ Antibodies
☒	☐ Eukaryotic cell lines
☒	☐ Palaeontology and archaeology
☒	☐ Animals and other organisms
☒	☐ Human research participants
☒	☐ Clinical data
☒	☐ Dual use research of concern

Methods

n/a	Involved in the study
☒	☐ ChIP-seq
☒	☐ Flow cytometry
☒	☐ MRI-based neuroimaging
